# Barriers of Adherence among Palestinian Healthcare Professionals towards the Protocol of Health Education and Counselling on Healthy Behaviours for Non-Communicable Diseases

**DOI:** 10.4314/ejhs.v31i1.9

**Published:** 2021-01

**Authors:** Ahmed Hassan Albelbeisi, Ali Albelbeisi, Abdel Hamid El Bilbeisi, Mahmoud Taleb, Amirhossein Takian, Ali Akbari-Sari

**Affiliations:** 1 Department of Health Management and Economics, School of Public Health, Tehran University of Medical Sciences, International Campus (TUMS-IC), Tehran, Iran; 2 In-service Health Education, European Gaza Hospital, Ministry of Health, Palestine; 3 Department of Clinical Nutrition, Faculty of Pharmacy, Al Azhar University of Gaza, Palestine; 4 Faculty of Pharmacy, Al Azhar University of Gaza, Palestine; 5 Department of Global Health and Public Policy, School of Public Health, Tehran University of Medical Sciences, Tehran, Iran; 6 Health Equity Research Centre (HERC), Tehran University of Medical Sciences, Tehran, Iran

**Keywords:** Adherence, barriers, non-communicable diseases, package of essential non-communicable diseases (PEN), protocol

## Abstract

**Background:**

Despite the huge numbers of the universally produced and employed protocols, the adherence with them is still low to moderate in the healthcare settings. This study was employed to assess the attitudes of Palestinian healthcare professionals in Gaza Strip to health education and counseling on healthy behaviours protocol (WHO-PEN Protocol 2), for patients with non-communicable diseases in the Ministry of Health primary healthcare centers.

**Methods:**

This cross-sectional study was conducted with a census sample of all governmental family physicians and nurses (n=175). The study questionnaire was developed based on Cabana theoretical framework. The Arabic version questionnaire was developed based on the cross-cultural adaptation framework. The psychometric properties of the Arabic version questionnaire was finally evaluated.

**Results:**

The psychometric properties of the Arabic version questionnaire showed good construct validity and internal consistency reliability. The overall adherence level to WHO-PEN Protocol 2 was 70.0, SD=6.9. The main perceived barriers were lack of incentive, patients' factors, and lack of time. In general, most of healthcare professional respondents had a positive attitude toward the protocol, but this attitude was not predictor to protocol adherence.

**Conclusion:**

The good validity and reliability of the questionnaire can provide support for the accuracy of the study results. Varied implementation strategies targeting the major barriers derived from the study are extremely required for addressing the lack of incentives, patients' factors and time constraints.

## Introduction

Non-communicable Diseases (NCDs) are generated by a combination of genetic, physiological, environmental and behaviors factors (1). The World Health Organization (WHO) valued that the four main types of NCDs were accountable for 41 million deaths yearly and reason for 71% of all deaths globally (2). Most of NCDs deaths (74%) and the majority of premature deaths (82%) occur in low- and middle-income states, where this public health crisis is especially challenging due to the severe social and economic conditions already faced by these states (3). Similar to other low- and middle-income countries, Palestine is undergoing a rapid epidemiological transition, with increasing the burden of chronic diseases (4). These days, Palestinian population has witnessed a notable change in lifestyles, nutritional behaviors and environmental surroundings (5). In addition, about two-thirds of elderly Palestinian suffers from NCDs (5). In Palestine, cardiovascular diseases remains the first leading cause of death, cancer was the second leading cause of death, and complications of diabetes came in the fourth rank (6).

The WHO developed package of essential NCDs interventions (WHO-PEN) for primary healthcare in low-resource settings. The WHO-PEN is the minimum standard for NCDs to strengthen national capacity to integrate and scale up care of NCDs in primary healthcare settings (7). The implementation tools of WHO-PEN including protocols to deal with different types of NCDs (7). An important way to control NCDs is to focus on reducing the risk factors associated with these diseases (8). The main types of NCDs share common behavioral risk factors (3). There is widespread scientific and public health policy agreement that these factors contribute significantly to chronic diseases morbidity and mortality (3). The WHO-PEN Protocol 2 is important tool in the implementation of WHO-PEN interventions, which focus on health education and counseling on healthy behaviors, to be applied for all NCDs patients. It is included instructions to educate patient on: Take regular physical activity, eat heart “healthy diet”, stop tobacco and harmful use of alcohol, and attend regular medical follow-up (7). In 2013, the Palestinian MOH introduced the WHO-PEN interventions for primary healthcare, which including, the training of staffs, adjusting record-keeping and the health information system, and support supervision. These interventions have delegated most NCDs management responsibilities to general physicians and nurses working in the PHCs (9).

The attention to the protocols as a significant knowledge translation tools has been increasing in the past decades (10). They are known as tools for development of evidence based medicine, are useful for reinforcing the quality of services, improve clients outcomes, and contain the costs (10). Although the huge numbers of the universally produced and employed protocols, the adherence with them is still low to moderate in the healthcare settings (11). Poor compliance to the protocols can harm patient safety, squandering resources, and lead to poor health outcome (12). The protocol implementation is a complex process affected by a lot of factors such as healthcare provider behavior, the protocol itself, and the method of implementing the recommendations (13). Cabana and his colleagues (13), established a framework, which includes seven wide-ranging barriers that are classified into three main categories: Knowledge related barriers, attitude-related barriers, and behavior-related barriers (13). The appropriate study of the factors delaying the healthcare professionals from being able to implement the protocols is the first step toward enhancing the adherence to protocols (14). This study was employed to assess the level and the major barriers of adherence to the WHO-PEN Protocol 2 for patients with NCDs.

## Methods

**Study design and setting**: This cross-sectional study was conducted with a census sample of all governmental family doctors and nurses (n=175) (15), who worked with NCDs patients in the fifty-two MOH-PHCs in the five governorates of Gaza Strip, Palestine (16). The total number of doctors and nurses working with NCDs patients in MOH-PHCs was 123 and 52, respectively (15). All healthcare professionals with minimum one-year working experience were included in the present study.

**Questionnaire development**: Based on the Cabana framework (13), the questionnaire was designed after revising the previous appropriate questionnaires (17,18,19). Most of the appropriate items measuring the structures of Cabana framework were revised and considered. A preliminary questionnaire with 50 items to assess the barriers of adherence to WHO-PEN Protocol 2, twelve items to assess the healthcare professionals' demographic and background. Also, 23 recommendations derived from the WHO-PEN Protocol 2 were included to measure the adherence level. Furthermore, a 5-points Likert scale was used for response categories.

**Translation and validation of the questionnaire**: The cross-cultural guideline process was employed in translation of the questionnaire (20). The questionnaire was translated into Arabic language on the five-step process (forward translation to Arabic by two Arabic native language translators, backward translation to English by two English native language translators, experts' committees review, pre-test, final Arabic version). Face and content validity were checked for the final Arabic draft questionnaire, independently validated by eight experts (researchers, academics, health experts, head nurse, and family doctors). Content validity index was calculated to rate the relevance of the questionnaire items (21). All items were rated as relevant with scores over 0.82. Minor changes in the language and the construction of the main recommendations items were done based on the agreement among the author (AHA), two family doctors and one head nurse. Then, the questionnaire was piloted among 30 of the eligible healthcare professionals. The results of the pilot study showed a good overall Cronbach's alphas of 0.84.

**Data collection**: The data was collected using an interview-based questionnaire, by five qualified data collectors (during September 2019 to January 2019), who were given explanation and training by the researcher about the study scope and purposes.

**Data analysis**: Statistical analysis was performed using SPSS version 22. The characteristics of the sample were described using descriptive statistics. Frequencies and percentages were used to described categorical variables, whereas means and standard deviations (SD) were used to represent continues variables. We tested the mean score differences between governorates with ANOVA test. P value less than 0.05 was considered as statistically significant.

**Construct validity**: Factor analysis was performed to examine the construct and underlying factor structure of the questionnaire. Extraction was done by using Principal Components Analysis with a Varimax rotation (22). The adequacy of data was evaluated based on the value of Kaiser-Meyer-Olkin (KMO) and Bartlett's test (22). The Kaiser Criterion with eigenvalues of ≥ 1 (23) was used to identify the number of extracted factors. Finally, factor loads under 0.30 were excluded (23).

**Internal consistency reliability**: Internal consistency reliability of the questionnaire was measured by calculating Cronbach's alpha coefficient of the overall questionnaire, and the identified domains with score over than 0.70 were considered good, reflecting the internal correlation between items of the same area (23). In addition, reverse coding was conducted to make sure that a higher score always means a more positive response.

**Ethical issues**: The study protocol was approved by the Ethics Committee of Tehran University of Medical Sciences (Code: IR.TUMS.REC.1398.349) and by the Palestinian Health Research Council (Helsinki Ethical Committee of Research PHRC/HC/599/19).

## Results

**Respondent's characteristics**: Table 1 shows the characteristics of the study participant's. From the 175 eligible participants, 160 replied to the interview, yielding a response rate of 91.4%. Approximately, half of the participants (55.6%) were males, and they had bachelor degree (78.1%). The mean age of the healthcare providers was 44.9, SD = 7.45 years, the mean of total years' experience (17.9, SD = 6.4) year, and the current work experience in centers (7.6, SD = 5.1) years.

**Table 1:** The characteristics of the study participants

Variables		N=160	Percentage %
Sex	Male	89.0	55.6
	Female	71.0	44.4
Age (years)	< 35	22.0	13.8
M±SD: 44.90±7.47	35 to 44	46.0	28.7
	45 to 60	92.0	57.5
Qualifications	Diploma	21.0	13.1
	Bachelor	125	78.1
	Postgraduate	14.0	8.8
Specialization	Medicine	108	67.5
	Nursing	52.0	32.5
Positions	Practitioners	124	77.5
	Manager	36.0	22.5
Total work experience (years)	≥ 5 years	2.0	1.3
M±SD: 17.90±6.44	6 to 10 years	17.0	10.6
	11 to 20 years	67.0	60.6
	> 20 years	44.0	27.5
Current work experience (years)	≥ 5 years	67.0	41.9
M±SD: 7.58±5.15	6 to 10 years	62.0	38.8
	11 to 20 years	28.0	17.5
	> 20 years	3.0	1.90

Health workers regular trained on NCDs (Total centers)		15 of 52	28.8

Health workers trained one-time on NCDs (Total centers)		37 of 52	71.2

Data are expressed as M±SD for continuous variables and as percentage for categorical variables. NCDs: Non-communicable diseases; M±SD: Mean ± Standard Deviation

**Psychometric properties of the questionnaire**: Table 2 shows that the KMO measure was 0.72, which represents the adequacy of the sample size for factor analysis (24). The Bartlett's test demonstrated that the inter item correlations were significant (χ2 = 4169.01; df = 1225; P < 0.001). In addition, the principal component analysis indicated a total of ten factors, with eigen values of ≥ 1 which accounted for 64.49% of the variance. The new factor structure seems to be reasonable, and reflects a more conceptual construct over this data set.

**Table 2:** Exploratory factor analysis of the overall questionnaire

Domains	Items	Eigenvalues	Variance*	Alpha**
F1: Agreement	Q46, Q47, Q48, Q49, Q50, Q51, Q52, Q53, Q54, Q55, Q56	10.45	20.91	0.84
F2: Knowledge and skills	Q36, Q38, Q39, Q40, Q41, Q42, Q43, Q44, Q45, Q57, Q59	4.58	9.16	0.82
F3: Lack of resources	Q37, Q71, Q72, Q73, Q74, Q75	4.35	8.71	0.79
F4: Motivation / inertia of previous practice	Q58, Q60, Q61, Q62, Q63, Q64	2.48	4.97	0.80
F5: Lack of incentives	Q76, Q77, Q78, Q79	2.30	4.06	0.89
F6: Lack of time	Q65, Q66, Q67, Q68	2.09	4.19	0.65
F7: Patient factors	Q84, Q85	1.70	3.41	0.85
F8: Protocol clarity	Q80, Q81	1.63	3.27	0.74
F9: Organizational support	Q69, Q70	1.60	3.20	0.65
F10: Protocol trustworthiness	Q82, Q83	1.29	2.59	0.74

Overall			64.49	0.90

Q: Question (Questions 36 to 85, numbers of total questions = 50). Factor analysis, Extraction method: Principal component analysis; Rotation method: Varimax with Kaiser Normalization

* Explained variance

** Cronbach α

**Internal consistency and reliability**: After conducting the construct validation, the Cronbach's alpha was computed (Table 2). The overall Cronbach's alpha was 0.90, which indicates a good correlation and consistency between the items and the questionnaire. In the 10-factor solution, alphas ranged from 0.65 to 0.89 for the different factors.

**Adherence to health education and counseling on healthy behaviors**: Table 3 displays that the adherence mean score across the key recommendations of WHO-PEN Protocol 2 was 70.0, SD = 6.9. The higher adherence score (69.6%) was for “the adherence to treatment” while the “stop smoking” was the lowest (13.8%). In addition, half of healthcare providers were always and often educate patients to take regular physical activity; and 34.8% always and often educate patients to eat heart healthy diet. Furthermore, 48.2% of healthcare providers reported that they always and often adhere to educate the patient about the key recommendations of the protocol; and 17.3% of the health care providers reported that they rarely and never adhere to educate the patient about the key recommendations of the protocol.

**Table 3:** The adherence mean score, across the key recommendations of WHO-PEN Protocol 2

Key recommendations	PHCs-MOH (N=160)		

	Always and often N (%)	Sometimes N (%)	Rarely and never N (%)
Take regular physical activity			
Progressively increase physical activity to moderate levels (such as brisk walking); at least 150 minutes per week.	60.0 (37.5)	78.0 (48.8)	22.0 (13.8)
Control body weight and avoid overweight by reducing high calorie food and taking adequate physical activity.	100.0 (62.5)	42.0 (26.3)	18.0 (11.3)
Percentage	50.0	37.5	12.5

Eat healthy diet			
Restrict salt to less than 5 grams (1 teaspoon) per day.	48.0 (30.0)	75.0 (46.9)	37.0 (23.1)
Reduce salt when cooking, limit processed and fast foods.	101.0 (63.1)	43.0 (26.9)	16.0 (10.0)
5 servings (400–500 grams) of fruits and vegetable per day.	46.0 (28.7)	87.0 (54.4)	27.0 (16.9)
1 serving is equivalent to 1 orange, apple, mango, banana or 3 tablespoons of cooked vegetables.	70.0 (43.8)	79.0 (49.4)	11.0 (6.9)
Limit fatty meat, dairy fat and cooking oil (less than two tablespoons per day).	64.0 (40.0)	74.0 (46.3)	22.0 (13.8)
Replace palm and coconut oil with olive, soya, corn, rapeseed or safflower oil.	36.0 (22.5)	85.0 (53.1)	39.0 (24.4)
Replace other meat with chicken (without skin).	25.0 (15.6)	69.0 (43.1)	66.0 (41.3)
Percentage	34.8	45.7	19.5

Stop tobacco			
Encourage all non-smokers not to start smoking.	4.0 (2.5)	28.0 (17.5)	128.0 (80.0)
Strongly advise all smokers to stop smoking and support them in their efforts.	41.0 (25.6)	84.0 (52.5)	35.0 (21.9)
Individuals who use other forms of tobacco should be advised to quit.	21.0 (13.1)	85.0 (53.1)	54.0 (33.8)
Percentage	13.8	41.0	45.2

Adherence to treatment			
Teach the patient how to take prescribed medicines at home.	151.0 (94.4)	9.0 (5.6)	0.0 (0.0)
Explain the difference between medicines for long- term control (e.g. Blood pressure) and medicines for quick relief (e.g. For wheezing).	59.0 (36.9)	98.0 (61.3)	3.0 (1.9)
Tell the patient the reason for prescribing the medicine/s.	145.0 (90.6)	13.0 (8.1)	2.0 (1.3)
Show the patient the appropriate dose.	151.0 (94.4)	8.0 (5.0)	1.0 (0.6)
Explain how many times a day to take the medicine.	152.0 (95.0)	7.0 (4.4)	1.0 (0.6)
Label and package the tablets.	65.0 (40.6)	50.0 (31.3)	45.0 (28.1)
Check the patient's understanding before the patient leaves the health center.	38.0 (23.8)	82.0 (51.2)	40.0 (25.0)
Explain the importance of keeping an adequate supply of the medications.	104.0 (65.0)	50.0 (31.3)	6.0 (3.8)
Explain the importance of the need to take the medicines regularly as advised even if there are no symptoms.	138.0 (36.3)	14.0 (8.8)	8.0 (5.0)
Percentage	69.6	23.0	7.4

Overall percentage	48.2	34.5	17.3
M±SD: 70.0±6.9; Median: 70.4			

Data are expressed as M±SD for continuous variables and as percentage for categorical variables. PHCs-MOH: Primary Health Care Centers - Ministry of Health; M±SD: Mean ± Standard Deviation

## The Perceived Barriers of Adherence to Health Education and Counseling on Healthy Behaviors

**Knowledge and skills**: Table 4 shows that the mean score of knowledge and skills was 70.92%, SD =10.56. A significant difference of knowledge and skills was found in the five Gaza Strip governorates, P-value = 0.014. The highest mean score among the five governorates was in Rafah governorate (76.5% SD = 8.5), and the lowest was in North Gaza governorate (65.4%, SD = 9.3). In addition, the vast majority (84.3%) of PHCs physicians and nurse claimed that they had adequate knowledge and skills to implement the key recommendations of the WHO-PEN Protocol.

**Table 4:** The perceived barriers of adherence to health education and counseling on healthy behaviors (WHO-PEN Protocol 2)

Barriers	5 and 4 N (%)	3 N (%)	2 and 1 N (%)	Mean± SD	F	P value
Knowledge and skills	97.0 (60.62)	23.0 (14.37)	40.0 (25.00)	70.92±10.56	3.22	0.014
Agreement	101.0 (63.12)	35.0 (21.87)	24.0 (15.00)	75.17±10.05	8.97	0.001
Motivation / inertia of previous practice	69.0 (43.12)	51.0 (31.87)	40.0 (25.00)	65.77±9.11	4.15	0.003
Lack of time	67.0 (41.87)	57.0 (35.62)	36.0 (22.50)	65.37±13.04	0.564	0.689
Organizational constraints	98.0 (61.25)	33.0 (20.62)	29.0 (18.12)	74.06±17.99	2.21	0.070
Lack of resources	95.0 (59.37)	33.0 (20.62)	32.0 (20.00)	72.56±9.91	7.30	0.001
Lack of incentives	33.0 (20.62)	42.0 (26.25)	85.0 (53.12)	49.50±17.24	1.31	0.266
Protocol trustworthiness	134.0 (83.75)	17.0 (10.62)	9.0 (5.62)	84.87±16.44	2.99	0.020
Protocol clarity	120.0 (75.00)	32.0 (20.00)	8.0 (5.00)	80.93±16.08	1.17	0.324
Patient factors	37.0 (23.12)	53.0 (33.12)	70.0 (43.75)	53.81±21.00	1.22	0.303

The differences between means were tested by using One-way ANOVA. P value less than 0.05 was considered as statistically significant. 5, 4, 3, 2, and 1 indicate respondents strongly agree, agree, are neutral, disagree, and strongly disagree, respectively

**Agreement**: Table 4 shows that the mean score of healthcare providers attitude to the WHO-PEN Protocol 2 in general was 75.17%, SD = 10.05. There were significant differences in attitude towards WHO-PEN Protocol 2 among healthcare providers in the five Gaza Strip governorates, P-value = 0.001. The highest mean score among the five governorates was in Rafah governorate (82.9%, SD = 6.1), and the lowest was in North Gaza governorate (66.7%, SD = 6.7). Furthermore, most of healthcare providers (75.6%) agreed that the protocol is suitable educational tool. The majority of healthcare providers (69.4%) agreed that the implementation of WHO-PEN Protocol 2 could improves the quality of clinical practice. The vast majority of healthcare providers (85.6%) agreed that implementation of the protocol can reduce healthcare costs.

**Motivation/inertia of previous practice**: Table 4 shows that the mean score of the motivation/inertia of previous practice was 65.77%, SD = 9.11. Significant difference of motivation was found in the five governorates of Gaza Strip, P = 0.003. The highest mean score among the five governorates was in Khan-Yunis governorate (70.7%, SD = 10.6), and the lowest was in Deir Al Balah (61.7%, SD = 11.9). Moreover, 61.3% of healthcare providers reported that they were able to cope with the change toward implementation of the WHOPEN Protocol 2 recommendations.

**Lack of time**: Table 4 shows that the mean of score of lack of time was 65.37%, SD = 13.04. No significant variations were found in perceiving lack of time in the five governorates of Gaza Strip, P-value = 0.689. The highest mean score among the five governorates was in Deir Al Balah governorate (68.4%, SD = 13.1), and the lowest was in Gaza governorate (64.1%, SD = 14.0). Additionally, 52.5% of healthcare providers reported that adherence to WHO-PEN Protocol 2 is time consuming.

**Organizational constraints**: Table 4 shows that the mean score of organizational constraints was 74.06%, SD =17.99. There were insignificant variations in perceiving organizational constraints in the five governorates of Gaza Strip, P-value = 0.070. The highest mean score among the five governorates was in Khan-Yunis governorate (81.0%, SD = 23.3), and the lowest was in Deir Al Balah governorate (69.7%, SD = 20.6). Furthermore, 68.1% of healthcare providers claimed that the current job description facilitates the implementation of WHO-PEN Protocol 2. Only 54.3% of healthcare providers announced that the current management is supporting the implementation of key recommendations of WHO-PEN Protocol 2.

**Lack of resources**: Table 4 shows that the mean score of lack of resources was 72.56%, SD = 9.91. A significant difference in perceiving lack of resources was found in the five governorates of Gaza Strip, P-value = 0.001. The highest mean score among the five governorates was in Khan-Yunis governorate (77.9%, SD =11.7), and the lowest was in Gaza governorate (68.7%, SD = 8.8). In addition, 83.8% of healthcare providers claimed that the medications included for NCDs management are in frequent shortage in this clinic, and 81.8% of healthcare providers reported that the laboratory tests for diagnosis and follow-up of NCDs patients are in frequent shortage.

**Lack of incentives**: Table 4 shows that the mean score of lack of incentives was 49.50% SD = 17.24. There were insignificant variations in perceiving lack of incentives in the five governorates of Gaza Strip, P-value = 0.266. The highest mean score among the five governorates was in Gaza governorate (52.0%, SD = 18.9), and the lowest was in Rafah governorate (43.5%, SD = 17.0). Moreover, only 11.2% of healthcare providers stated that the current monthly salary motivated them to adherent to WHO-PEN Protocol 2, and 21.9% of healthcare providers reported that they received acknowledgment from top management as long as they follow the protocol recommendations.

**Protocol trustworthiness**: Table 4 shows that the mean score of protocol trustworthiness was 84.87%, SD = 16.44. Perception of protocol trustworthiness is statistically significant in the five governorates of Gaza Strip, P-value = 0.020. The highest mean score among the five governorates was in Deir Al Balah governorate (92.2%, SD = 14.0), and the lowest was in North Gaza governorate (79.7%, SD = 19.6). Furthermore, the vast majority (85.0%) of healthcare providers stated that they agreed that the WHO-PEN Protocol 2 was developed by qualified and knowledgeable experts.

**Protocol clarity**: Table 4 shows that the mean score of protocol clarity was 80.9%, SD =16.08. There was a sinsignificant difference in attitude towards protocol clarity in the five governorates in Gaza Strip, P-value = 0.324. The highest mean score among the five governorates was in Rafah (90.0%, SD = 9.4), and the lowest was in Khan-Yunis governorate (78.8%, SD = 17.8). Additionally, the vast majority (81.2%) of healthcare providers claimed that the WHO-PEN Protocol 2 is well organized and the key recommendations are clear and specific.

**Patient's factors**: Finally, Table 4 shows that the mean score of the patient factors was 53.81%, SD = 21.0. A statistically insignificant variation was found in the five governorates of Gaza Strip, P-value = 0.303. The highest mean score among the five governorates was in Khan-Yunis (59.3%, SD = 21.1), and the lowest was in Gaza governorate (49.7%, SD = 20.4). Moreover, only 21.9 of healthcare providers agreed that patients wanted physicians and nurses to conform to the WHO-PEN Protocol 2.

## Discussion

The analysis of the developed questionnaire used to assess the barriers of adherence to the WHO-PEN Protocol 2 demonstrated very good psychometric properties. It has a good construct validity and internal consistency reliability. To the best of our knowledge, this is the first study in Gaza Strip, which assess the factors hindering the adherence to WHO-PEN Protocol 2. The general adherence to the protocol is (70.0, SD = 6.9). Teach the patients to attend regular medical followup is the highest (69.6%) adherence by healthcare providers among the key recommendations of the protocol, followed by attend regular physical activity (50%), eat heart healthy diet (34.8%). Teach the patients to stop tobacco is the lowest adherence (13.8%) by healthcare providers among the key recommendations of the protocol. A study conducted in Sri Lanka demonstrated that (41.3%) of medical officer advised NCDs patients to change their life styles (25). In Saudi Arabia, family medicines adherence to educate NCDs patients to attend regular physical activity varied between 66% to 96% for NCDs patients (26). In UK, only 11% of physician advised diabetic patients to change life style (27). A study conducted in China demonstrated that 28.3% of physicians advised hypertensive patients to lifestyle modifications (28). In West Bank, Palestine, there were variations between healthcare professionals in implementation health education and counseling on healthy behaviors for cardiovascular diseases. Only 31% of physicians and 13.2% of nurses implemented counseling on medications, 20% of physicians and 72.1% of nurses implemented counseling on diet. About 13.8% of physicians and 66.2% of nurses implemented counseling on physical activity, and 3.4% of physicians and 35.3% of nurses implemented counseling on tobacco cessations (29). In Gaza Strip, Palestine, Radwan et al. demonstrated that only 15.1% of MOH-PHCs and 51.2% of the UNRWA healthcare professionals were adhere to start therapy with education, diet and exercises for diabetes mellitus patients (15).

Although the protocols development for healthcare providers has acquired momentum in last years, this does not indicate that the key recommendations included in the protocols are really implemented (14). In general, adherence to protocols range from low to moderate (13). Several systematic reviews reported that most of the adherence promoting interventions had low to moderate effects (10,30). Such inadequate effects might be due to the inappropriate use of the behavioral and organizational theories as a guide for promoting the adherence (31).

Generally, the study demonstrated that there were many significant variations in perceiving the barriers among healthcare providers in PHCs of the five Gaza Strip governorates. There were significant differences in knowledge and skills, agreement and motivation/inertia of previous practice, lack of resources and protocol trustworthiness. Furthermore, there were statistically significant differences in means between the five Gaza Strip governorates in term of attitude/agreement. Firstly, the attitudinal barriers toward the protocol adherence can be excluded since the most of healthcare providers had a positive attitude toward the protocol. Most of healthcare providers (75.6%) agreed that the protocol is suitable educational tool. The majority of healthcare providers (69.4%) agreed that the implementation of WHO-PEN Protocol 2 can improve the quality of clinical practice, 84.3% of PHCs physicians and nurses' respondents claimed that they had adequate knowledge and skills to implement the key recommendations of the WHO-PEN Protocol 2. A possible explanation of the significant differences between the five Gaza Strip governorates that healthcare professional skills in some governorates are poorly developed or underused (32). Inequitable distribution of health centers and healthcare professionals within Gaza Strip governorates, favoring Gaza Strip central areas, contributes to health inequity (33).

Additionally, the most perceived barriers in PHCs were lack of incentives, patients' factor and lack of time. The lack of incentives was defined as one of the barriers for implementing the NCDs guidelines (15,34). Our study demonstrated that only 11.2% of healthcare providers stated that the current monthly salary motivated them to adhere to WHO-PEN Protocol 2. Radwan et al. (15), described lack of incentives as the most frequently perceived barrier to adherence to diabetes mellitus clinical practice guidelines in Gaza Strip. Moreover, a recent systematic review demonstrated that the pay for performance have changeable effects on physician behavior (35). A possible explanation for this results is that the vast majority of the Palestinian governmental staff in Gaza Strip including MOH staff since 2006 did not receive their full salary and the government was not able to satisfy the healthcare professional because of low salaries (32). It appears reasonable to study what might motivate the healthcare providers before any application of protocol. After that, the Palestinian governmental payment way and the incentive strategies should be wisely revised and redesigned, taking into consideration financial and non-financial incentives.

Patient factors play a major role in hindering the adherence to WHO-PEN Protocol 2, only 21.9% of healthcare providers agreed that patients wanted physicians and nurses to conform with the WHO-PEN Protocol 2. Only 24.4% of healthcare providers claimed that patients are interested to be involved in care plan according to WHO-PEN Protocol 2.

In a study conducted in the West Bank PHCs, the healthcare professionals revealed that patient factors were one of the barriers to the implementation of PEN Protocols. In addition, the healthcare professionals claimed that NCDs patients do not come in the specified time for medical follow-up, usually in hurry, coming mainly to the health center to get their medications, ignoring doctors' advices, and do not cooperate with nurses (29).

Saillour-Glenisson et al. (36) also defined patients' resistance to the recommendations of the protocol as a factor affecting negatively the adoption of the protocol.

The lack of time as the third perceived barrier affected the healthcare professional's adherence to WHO-PEN Protocol 2. Only 24.4% of the doctors and nurses claimed that implementing the WHO-PEN Protocol 2 recommendations did not add extra burden to their assigned tasks, 22.5% of them declared that they did not reduce the consultation time for NCDs patients because of heavy workload. Lack of time and workload have been found to be challenges to guidelines implementation (15). Finally, in spite of the positive attitude towards the protocol among healthcare professionals, it was not a predictor of protocol adherence (37).

The findings of the study support the Cabana framework, as a model for assessing the protocols barriers. A possible limitation of the study is that it is based on self-reported data, which could lead to recall bias and social desirability bias.

In conclusion, the results of our study support Cabana theoretical framework, as a framework to assess the adherence barriers for protocols. The good validity and reliability of the questionnaire can provide support for study results. Varied implementation plans targeting the main barriers derived from the study are extremely required for addressing the lack of incentives, patient's factors and time constraints.
